# *Tobacco Induced Diseases *moves to BioMed Central

**DOI:** 10.1186/1617-9625-4-1

**Published:** 2008-07-31

**Authors:** J Elliott Scott

**Affiliations:** 1 University of Manitoba, Faculty of Dentistry, 780 Bannatyne Avenue, Winnipeg, MB, R3E 0W2, Canada

## Abstract

This Editorial marks the transfer of *Tobacco Induced Diseases *to BioMed Central's publishing platform.

## Editorial

Alongside this article, the first articles accepted by *Tobacco Induced Diseases *are published on BioMed Central's open access publishing platform. Until now, the journal has been hosted at the University of Manitoba in Winnipeg, Canada. The journal and its host society, the International Society for the Prevention of Tobacco Induced Diseases (ISPTID), have an interesting history [1]. Originally founded as a forum for exchange of information concerning a common interest in the prevention of disease induced by cigarette smoking, this society has transformed into an internationally recognized group of experts on international anti-smoking law implementation and enforcement efforts. In addition, we are now mounting an effort to extend our publication efforts into the areas of basic biological effects of tobacco smoke constituents. As a scientist who primarily undertakes research in this latter category, I find myself at the society meetings in a somewhat foreign environment. In the presence of policy and epidemiological experts and analysts, a basic biomedical scientist hears about duplicitous dealings of tobacco companies, shifts in cigarette use demographics, and, most importantly, real impacts, including fatal impacts, of smoking on populations and individuals. For a molecular researcher this is indeed new territory and has become an interesting experience, a foray out of the laboratory and maybe a chance to find the much sought-after relevance for all the work of students, technicians and collaborators. Indeed, the ISPTID has provided a growing international platform to explore many aspects of disease induced by tobacco smoke. For this reason, as well as to stress the emphasis on molecular research in the medical literature, we have expanded our editorial research board to include Xing Li Wang who brings scientific credibility in the area of vascular genetics, cell and molecular biology. Dr. Wang is Professor of Surgery in the Cardiothoracic Surgery Division of the Michael E. DeBakey Department of Surgery at Baylor College of Medicine and at the Texas Heart Institute at St. Luke's Episcopal Hospital in Houston, Texas.

New submissions to *Tobacco Induced Diseases *have reached a constant flow. Furthermore, with the move to BioMed Central, the material we accept for publication will be widely visible and fully indexed [2]. Past authors will benefit as well as the society executive has made the important decision to move all past issues of *Tobacco Induced Diseases *to BioMed Central, thus providing enhanced public access to this scientific information.

Forthcoming articles to be published in *Tobacco Induced Diseases *reflect the broad scope of the journal and emphasize the diversity of publication interests of the society and the Editorial Board. The highly original article by Georg Matt *et al. *on the impact of tobacco smoking on the asking price for used cars presents a psychological analysis of how buyers view the value of cars which have been exposed to tobacco smoke [3]. It finds that nonsmokers' cars brought a premium price in the used car market. Kazunari Satomura and colleagues discuss Japanese anti-smoking policies [4] – this is timely as the ISPTID society meets in Kyoto in September of 2008, and with a significant contingent of tobacco policy analysts attending Japanese tobacco control initiatives will receive a great deal of attention [5]. On a more fundamental level, Lennart Larsson and his research group present a unique analysis using sophisticated molecular approaches, specifically gas chromatography-tandem mass spectrometry, to measure the microbial load in cigarette tobacco and smoke [6]. Similarly, Azida Walker *et al. *present evidence that nicotine alters mitogenic potential in AR42J cells, a rat pancreatic tumor cell line which has implications for smoke-induced pancreatic cell damage [7]. Clearly, these are all significant and important observations of the effects and disease-causing potential of cigarette smoke on biological systems and its ability to induce serious health problems.

Finally we would like to express our thanks to all authors involved for their patience and understanding as a number of issues and delays have occurred with the move of *Tobacco Induced Diseases *to a new publishing platform. We hope readers, researchers and policy analysts will view *Tobacco Induced Diseases *as a channel for publications of their future findings.
